# An Invariant-Based Damage Model for Human and Animal Skins

**DOI:** 10.1007/s10439-016-1603-9

**Published:** 2016-04-11

**Authors:** Wenguang Li, Xiaoyu Y. Luo

**Affiliations:** 1 School of Engineering, University of Glasgow, Glasgow, G12 8QQ UK; 2 School of Mathematics and Statistics, University of Glasgow, Glasgow, G12 8QW UK

**Keywords:** Skin, Damage, Fibre orientation, Fibre orientation dispersion, Constitutive model, Inverse problem

## Abstract

Constitutive modelling of skins that account for damage effects is important to provide insight for various clinical applications, such as skin trauma and injury, artificial skin design, skin aging, disease diagnosis, surgery, as well as comparative studies of skin biomechanics between species. In this study, a new damage model for human and animal skins is proposed for the first time. The model is nonlinear, anisotropic, invariant-based, and is based on the Gasser–Ogden–Holzapfel constitutive law initially developed for arteries. Taking account of the mean collagen fibre orientation and its dispersion, the new model can describe a wide range of skins with damage. The model is first tested on the uniaxial test data of human skin and then applied to nine groups of uniaxial test data for the human, swine, rabbit, bovine and rhino skins. The material parameters can be inversely estimated based on uniaxial tests using the optimization method in MATLAB with a root mean square error ranged between 2.15% and 12.18%. A sensitivity study confirms that the fibre orientation dispersion and the mean fibre angle are among the most important factors that influence the behaviour of the damage model. In addition, these two parameters can only be reliably estimated if some histological information is provided. We also found that depending on the location of skins, the tissue damage may be brittle controlled by the fibre breaking limit (i.e., when the fibre stretch is greater than 1.13–1.32, depending on the species), or ductile (due to both the fibre and the matrix damages). The brittle damages seem to occur mostly in the back, and the ductile damages are seen from samples taken from the belly. The proposed constitutive model may be applied to various clinical applications that require knowledge of the mechanical response of human and animal skins.

## Introduction

The human skin not only has important protective functions against mechanical trauma such as friction, impact, pressure, cutting and shearing, but also plays a vital role in active thermo-regulation, wound-healing, and acts as the nonslip intermediate surface when one grips, lifts, or presses.^10^ The skin consists of three layers: the epidermis, the dermis, and subcutaneous tissues. The epidermis is the top renewable layer of 0.1–1.5 mm thickness. The dermis is the middle layer with 1–4 mm thickness,^46^ which has two sub-layers: the papillary layer and the reticular layer. The dermis consists of 77% collagen and 4% elastin (fat-free dry weight),^55^ vasculature, nerve bundles, hair follicles, veins and sweat glands. The subcutaneous tissue is underneath the dermis and with fat to store energy for the body.

The skin is in tension in normal physiological conditions and its tension level depends on individual maturation and aging, wound healing state, dysfunction or diseases such as the Ehlers–Danlos syndrome.^5^,^37^,^57^ Studying biomechanical property of human skin is useful in cosmetic product development, plastic surgery, surgical practice and skin disease pathology as well as artificial skin design.

**Figure 1:**
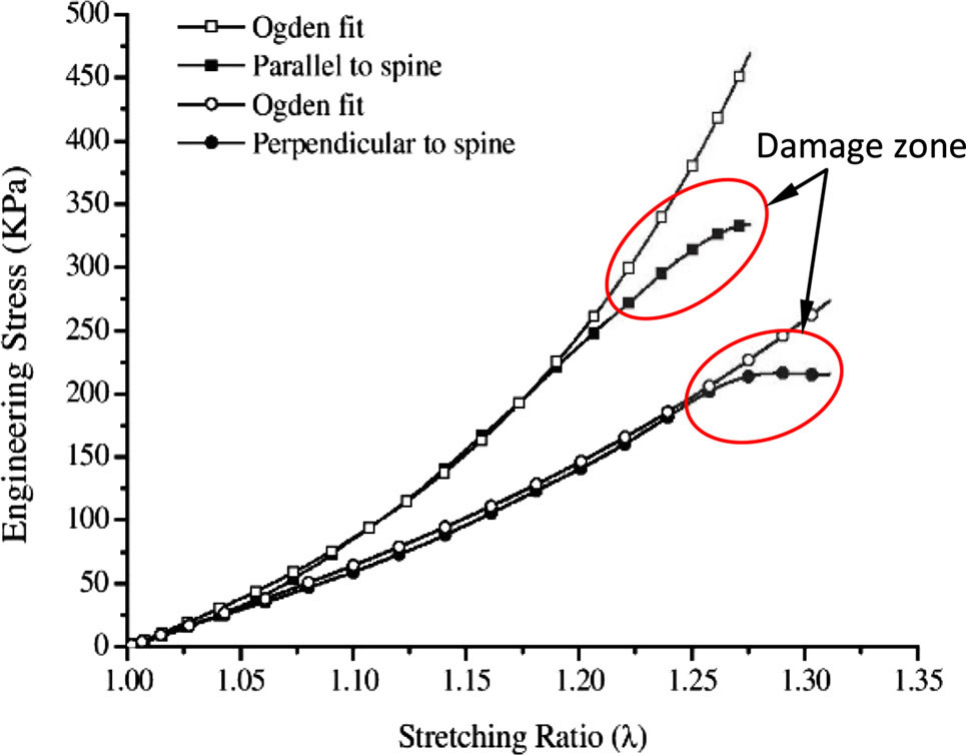
The stress-stretch curve of the skin samples harvested from the pig body along different directions, the *in vitro* tests were made at 2500 s^−1^ strain rate, two curves exhibit damage effect at a higher stretch, the model fails to fit the curves, the plot after Ref. 29.

Commonly used methods to identify skin biomechanical properties can be classified as *in vivo* and *in vitro* methods. *In vivo* methods are mainly adopted in daily clinical practice. With this method one can generate a stretch, shear, torsion, compression, indentation, or wave deformation, in order to compute the stress components or the material properties (e.g., Young’s moduli).^1^,^11^,^13^,^25^,^28^,^30^,^36^ However, with this approach, only up to 30% maximum extension can be obtained; the full nonlinear behaviour of skins cannot be accounted for in the damage models.^10^ *In vitro* methods provide alternative approaches, where skin samples are taken from the area of interest with the subcutaneous fat removed. These methods have been used to test biomechanical behaviour of skin, including its nonlinear, anisotropic, viscoelastic properties, and of skin damages at much higher strain rates than using *in vivo* methods. The specimens can be tested using one of the following devices: (1) uniaxial stretch device,^2^,^25^,^29^,^34^,^41^,^42^,^44^,^45^,^52^ (2) biaxial stretch device,^22^,^23^,^43^ (3) multi-axial device,^20^,^38^ and (4) bulge device.^9^,^48^ Of these, the uniaxial test is simplest and can capture the damage of testing specimens easily. A typical example is illustrated in Fig. 1, showing the damage of swine skin at the strain rate of 2500 s^−1^.^29^

Despite many experimental studies, to date, there is a lack of mathematical constitutive models for describing the highly nonlinear and anisotropic skin behaviour due to damage. Such constitutive laws are essential for understanding skin mechanics after trauma and injury, as well as for applications such as artificial skin design, skin aging, disease diagnosis, and surgical treatment. In this contribution, we aim to develop such a model for the first time, which can describe the biomechanical properties of damaged human and animal skins.

## Skin Histology and Constitutive Models

### Skin Histology

A detailed microscopy study^17^ showed that there are at least three collagen layers inside the dermis of human skin: a thin superficial layer with fine bundles of collagen, a middle layer, which makes up most of the dermal bulk, and a deep layer of fibres linking the skin to the superficial fascia. Changes from a stretch mainly occur in the middle layer, suggesting it is the major load-bearer.^17^ When relaxed, collagen fibres are un-stretched and wavy; under an extension, the collagen fibres are individually straightened until all of these are recruited.^17^ In human skin, the collagen fibres in the unstressed dermis layer are grouped in large and small bundles and there are connective fine and thread-like fibrils between them.^50^

**Figure 2:**
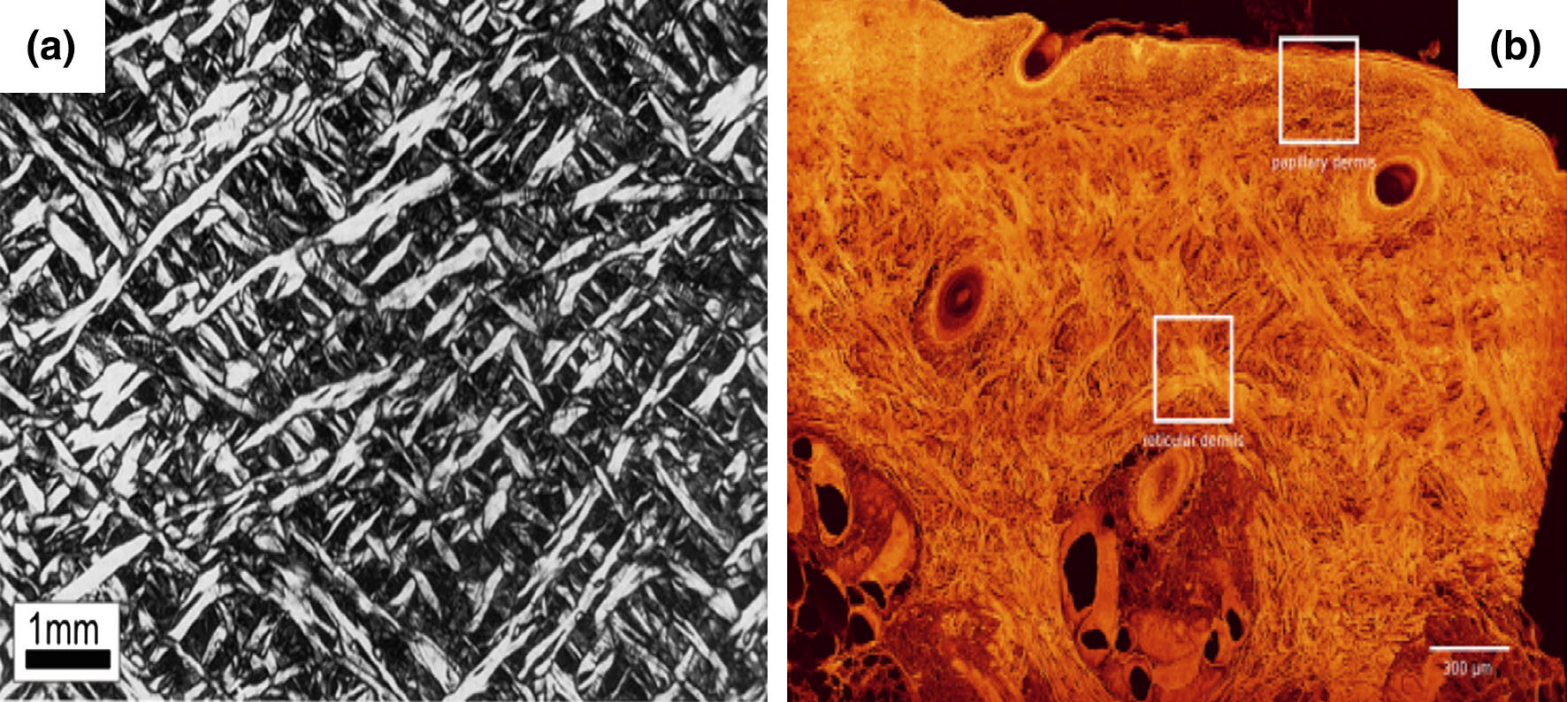
The collagen fibre (bright/bright red colour) network in the cross-section of the rhino and pig back skin dermis, (a) rhino in Ref. 44 (b) pig in Ref. 33.

Histology of skin samples from a rhinoceros back was observed by Shadwick *et al*.,^44^ who photographed the collagen fibre morphology and orientation using a stereoscope with polarising optics. It was shown that the fibres formed a cross-linked network in the cross-section of the dermis, see Fig. 2a. Recently, Jor *et al*.^21^ studied the collagen fibre structure in the abdomen of young swine using confocal laser scanning microscopy (CLSM) and image analysis. They found that the collagen fibres were grouped into large bundles in the reticular dermis and run between the epidermis and subcutaneous tissue (hypodermis) obliquely along two predominant orientations (Fig. 2b). It was also observed that a distinct lattice structure was apparent in all the sections perpendicular to the plane of the epidermis, and proposed a density distribution function to describe collagen fibre orientation. Using digital image analysis for the human dermis, Ni Annaidh *et al*. determined the collagen fibre orientation distributions in the plane parallel to the epidermis,^33^,^34^ and found that the mean fibre angle to Langer’s lines is 41°, and the fibre dispersion parameter is 0.14. They also proposed a 2D in-plane fibre orientation density function.

### Constitutive Models for the Skin

Ridge and Wright first proposed a one-dimensional exponential and power function load-extension models for animal skin^41^ using uniaxial load-extension tests of the human abdomen and foreman skins.^40^,^41^ 3D isotropic models were developed by using modified Mooney-Rivlin strain energy function for animal skin in Refs. 28,52,58 and human skin in Ref. 24.

Bischoff *et al*. performed finite element simulations using a constitutive model based on the entropy change upon stretching of long-chain molecules and the collagen network for rat skin.^4^ A collagen fibre recruitment model for rat skin was also proposed by Belkoff and Hutt.^3^ By adopting Veronda’s approach in Ref. 52 Groves *et al*. used an exponential strain energy functions for human and murine skins that included some fibre effects.^18^ However, the fibre angle and its dispersion effect are not taken into account explicitly. Jor *et al*. proposed a constitutive model for skin mechanics, which consists of homogenous matrix and anisotropic collagen fibres including the fibre orientation, dispersion, and the recruitment effect.^20^ More recently, Gasser *et al*. developed a structure-based strain energy function, also known as the Gasser–Ogden–Holzapfel (GOH) model, for arterial walls.^16^ This model also explicitly includes the collagen fibre orientation and the fibre dispersion:

1 $$ W = \frac{\mu }{2}\left( {I_{1} - 3} \right) + \frac{{k_{1} }}{{k_{2} }}\left\{ {\exp \left( {k_{2} \left[ {\kappa I_{1} + \left( {1 - 3\kappa } \right)I_{4} - 1} \right]^{2} } \right) - 1} \right\} , $$

where *μ*, *k*_1_ and *k*_2_ are the material constants, *κ* is the fibre orientation dispersion parameter, *I*_1_ is the invariant representing the squared stretch of the tissue, and *I*_4_ is the squared stretch along the fibre direction. This model has been successfully applied to the human skin by a number of groups.^33^,^47^,^49^ However, none of the aforementioned models has taken account of skin damage.

**Figure 3:**
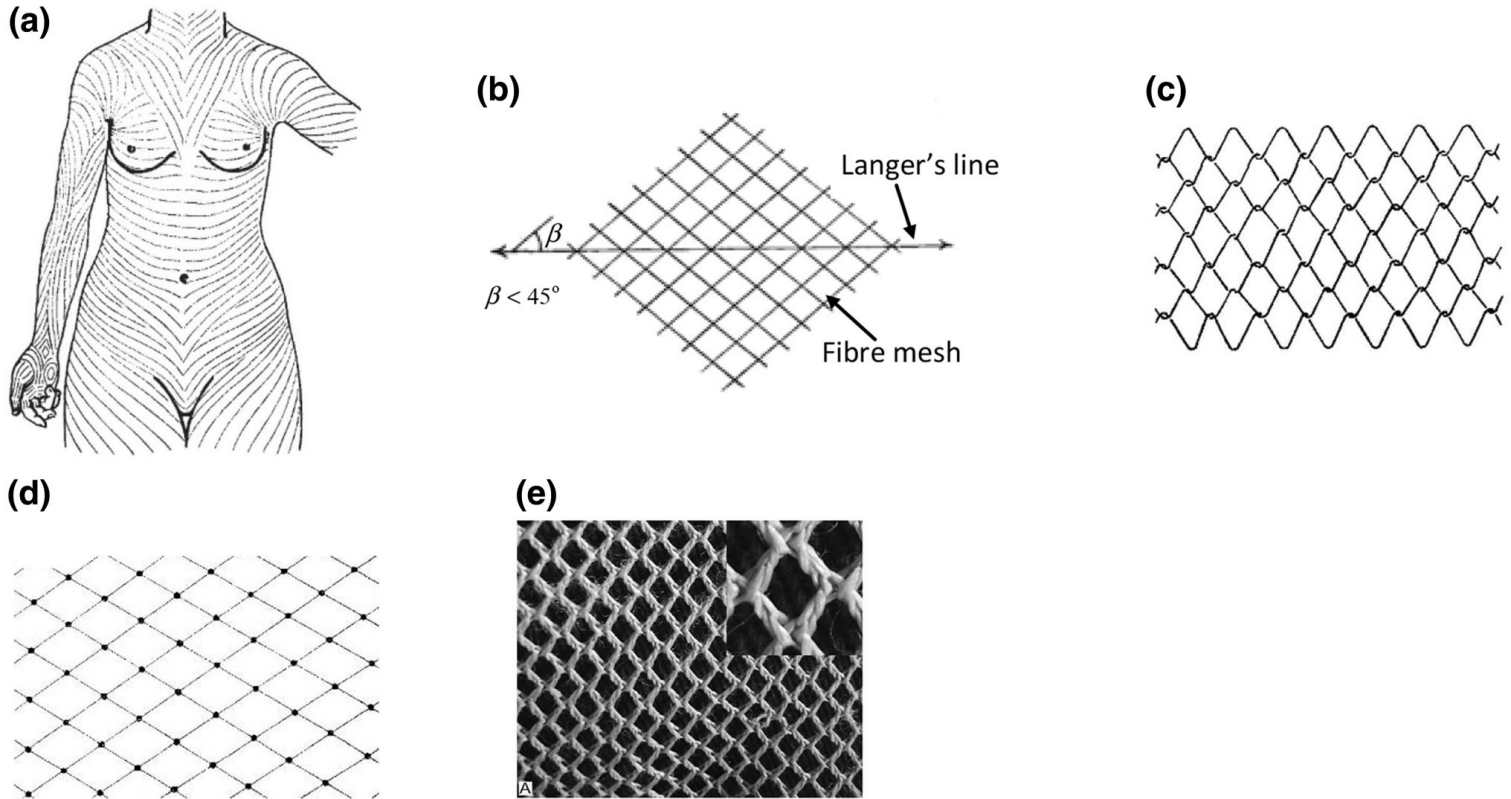
(a) The Langer’s lines,^8^ (b) the fibre mesh,^42^ (c) the 2D mobile fibre network proposed in Ref. 17 with slip joints, and (d) a 2D mobile fibre network with fixed joints,^50^ and (e) a woven 2D network in Ref. 39.

Damage of skin is often referred to as tissue softening, which is represented by the curvature change of the stress-stretch curve, as shown in Fig. 1. The stress field of the skin can be represented by a series of lines known as the Langer’s lines^8^ (Fig. 3a). Ridge and Wright found that the mean collagen fibre angle is more aligned in the direction of Langer’s lines, and put forward a fibre meshwork as shown in Fig. 3b.^42^ Based on this idea, Gibson *et al*. proposed a 2D interwoven network of collagen fibres as shown in Fig. 3c.^17^ In this meshwork, the fibres can rotate and slip at the joints, and when stretched all the fibres become parallel to the stretched direction. An alternative network was proposed by Tregear in Ref. 50 who assumed that the fibres are fixed but can rotate at the joints (Fig. 3d). Ribeiro *et al*. found that in the rat reticular dermis the collagen fibre bundles are in a woven 2D network in the observed plane similar to Fig. 3e.^39^

Although there are no damage models specifically for the skin, various damage models for other soft tissues have been developed; namely, for the porcine carotid,^15^ the human anterior rectus sheath,^31^ the vaginal tissue,^6^ and the human thoracic and abdominal aortas.^56^ For human atherosclerotic arteries, a cohesive fracture model was proposed to allow cracks to develop when the tensile strength reaches the maximum damage criterion.^12^ Other fibre and matrix structure-based strain energy functions, e.g.,^19^ have also been extended to include damage in Ref. 53,54 and other references shown in Ref. 26.

## A New Constitutive Model for Skin Damage

**Figure 4:**
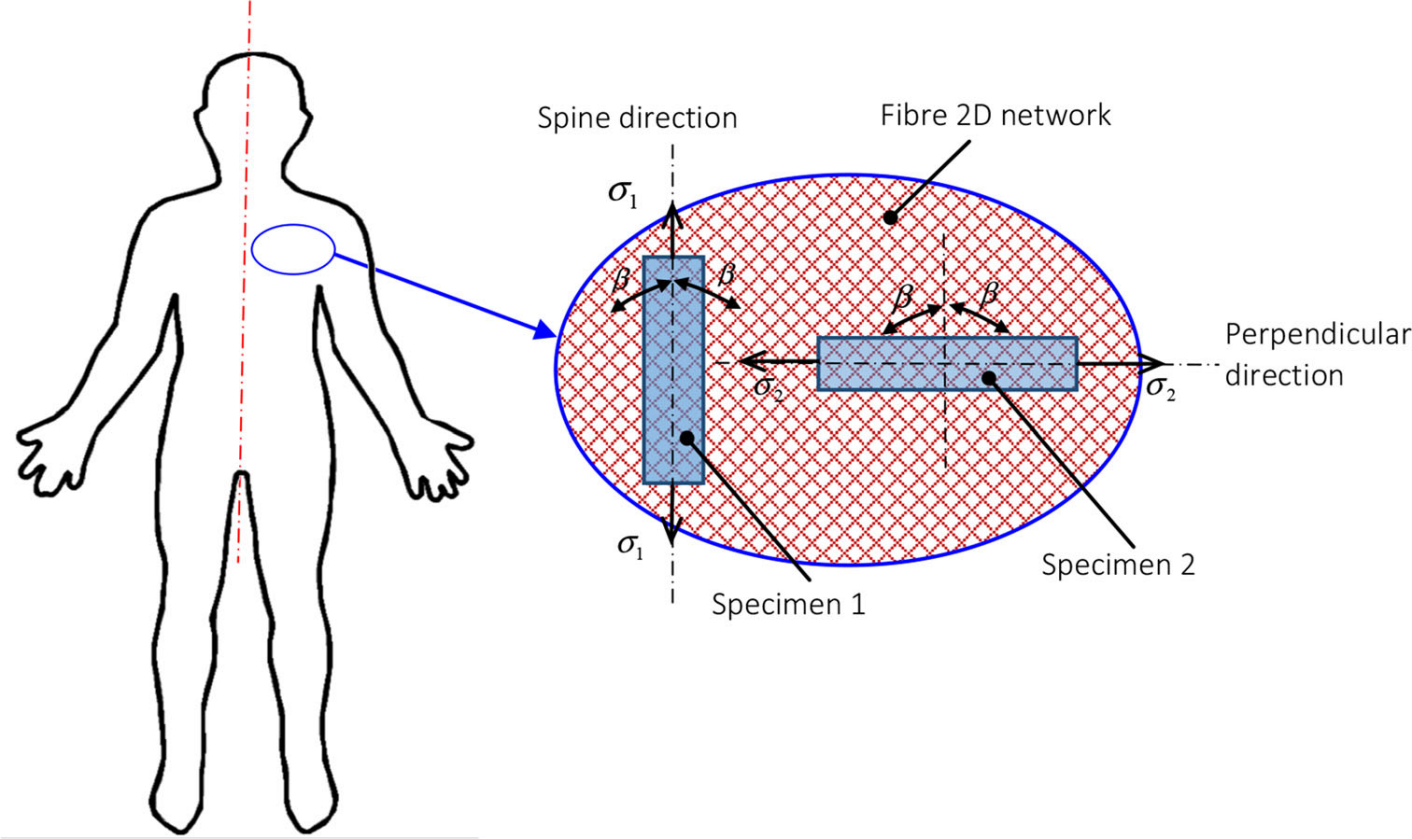
Locations of the skin of interest, with the two specimens harvested along the spine and the perpendicular directions, and the mean fibre orientation *β* is shown on the right.

In this paper, a new damage model for the skin is developed. In this model, the skin is assumed to be anisotropic, hyperelastic and incompressible, with two symmetric families of the collagen fibres embedded in a matrix. Each family of the fibres has a mean fibre angle of *β* with respect to the reference direction. We assume that the fibres have the same structure across the dermis depth, bounded by internal fibrils and form a meshwork with free rotation and slip. Although the matrix material consists of the epidermis and subcutaneous fat as well as the rest of the tissues in the dermis as shown in Fig. 4, it is assumed to be homogeneous in our continuum mechanics model.

Our model is essentially an extended GOH-type strain energy function, inspired by Volokh’s work for arterial walls,^53^,^54^

2 $$ W = \frac{\mu }{2}\left[ {\left( {I_{1} - 3} \right) - \frac{{\left( {I_{1} - 3} \right)^{m + 1} }}{{\left( {m + 1} \right)\left( {\zeta - 3} \right)^{m} }}} \right] + \frac{{k_{1} }}{{k_{2} }}\left\{ {\exp (k_{2} A^{2} ) - 1 - \frac{{2k_{2} A^{n + 2} }}{{\left( {n + 2} \right)\left( {\xi^{2} - 1} \right)^{n} }}} \right\}, $$

where $A = \lambda_{f}^{2} - 1$, with $\lambda_{f} = \sqrt {\kappa I_{1} + \left( {1 - 3\kappa } \right)I_{4} }$ is the fibre stretch, and *m*, *n*, *ξ* and *ζ* are the phenomenological parameters to describe the damage induced material softening. In particular, *m* and *ζ* are associated with the matrix damage; *m* specifies the sharpness of the stress-stretch curve when damage occurs, and *ζ* indicates the value of *I*_1_ when the matrix damage occurs, *n* and *ξ* are the corresponding parameters for the fibre damage; *n* is the counterpart of *m*, and *ξ* demonstrates when the fibres damage occurs in terms of *λ*_*f*_. If these parameters are chosen to be *ξ* = *ζ* =+∞ and *m* = *n* = 1, then the GOH model is recovered.

To determine the material parameters in (2), we make use of the data obtained from the uniaxial test protocol designed for both human and animal skins. The test is performed along the two orthogonal directions of the skin, namely, along and across the spine, as shown in Fig. 4. In the uniaxial test of the specimen 1, the stress component is measured ($\sigma_{1}^{ \exp }$). On the other hand, this can also be computed from (2),

3 $$ \sigma_{1} = \lambda_{1} \frac{\partial W}{{\partial I_{1} }}\frac{{\partial I_{1} }}{{\partial \lambda_{1} }} + \lambda_{1} \frac{\partial W}{{\partial I_{4} }}\frac{{\partial I_{4} }}{{\partial \lambda_{1} }} , $$

where *λ*_1_ *λ*_*t*_ *λ*_*h*_ = 1, *λ*_*t*_ = *λ*_*h*_, $I_{1} = \lambda_{1}^{2} + \lambda_{t}^{2} + \lambda_{h}^{2} ,$ $\, I_{4} = \lambda_{1}^{2} \cos^{2} \beta + \lambda_{t}^{2} \sin^{2} \beta ,$ *λ*_*t*_ and *λ*_*h*_ represent the transverse and thickness stretches, respectively. The stress component for the uniaxial test of specimen 2 can be similarly measured and computed.

The material constants are then estimated inversely by minimising the objective function

4 $$ F(\mu ,k_{1} ,k_{2} ,\beta ,\kappa ,m,n,\xi ,\zeta ) = \sum\limits_{i = 1}^{{n_{1} }} {\left( {\sigma_{1i} - \sigma_{1i}^{\exp } } \right)^{2} } + \sum\limits_{i = 1}^{{n_{2} }} {\left( {\sigma_{2i} - \sigma_{2i}^{\exp } } \right)^{2} } , $$

where *n*_1_ and *n*_2_ are the numbers of sample points in the test of specimens 1 and 2, respectively.

Similar to Ref. 27, the optimization process is carried out using the MATLAB (*lsqnonlin* function). The approximation between the measurement and the prediction is measured by the standard deviation error:

5 $$ \varepsilon = \frac{1}{{\sigma^{\text{mean}} }}\sqrt {\frac{{\sum\limits_{i = 1}^{{n_{1} }} {\left( {\sigma_{1i} - \sigma_{1i}^{\exp } } \right)^{2} + \sum\limits_{i = 1}^{{n_{2} }} {\left( {\sigma_{2i} - \sigma_{2i}^{\exp } } \right)^{2} } } }}{{n_{1} + n_{2} }}} , $$

normalised by the mean stress $\sigma^{\text{mean}} = {{\left( {\sum\limits_{i = 1}^{{n_{1} }} {\sigma_{1i}^{\exp } + \sum\limits_{i = 1}^{{n{}_{2}}} {\sigma_{2i}^{\exp } } } } \right)} \mathord{\left/ {\vphantom {{\left( {\sum\limits_{i = 1}^{{n_{1} }} {\sigma_{1i}^{\exp } + \sum\limits_{i = 1}^{{n{}_{2}}} {\sigma_{2i}^{\exp } } } } \right)} {(n_{1} + n_{2} )}}} \right. \kern-0pt} {(n_{1} + n_{2} )}}$. Specifically, for each specimen, we adopt the following procedure:

1. Specify the ranges of all the material parameters;
2. Normalise the parameters, and generate the initial guesses;
3. Set up the working variables, the error tolerance for the *lsqnonlin* function (10^−8^), the minimum and the maximum step changes of the variables (10^−4^ and 10^−3^), and the maximum number of the iterations (2 × 10^4^);
4. Calculate the objective function and update parameters by making use of the trust-region-reflective algorithm embedded in MATLAB;
5. If the objective function value is larger than the tolerance, go back to (4);
6. If any parameters are on a boundary, extend the boundary and go back to (2);
7. Use the optimal parameters in the model and compute the Cauchy stress at a stretch;
8. Output the model results, and compare with the experimental data.

We confirm that all the test data used in this paper are from the published references provided,^2^,^23^,^29^,^33^,^35^,^44^,^51^ and we have not harvested, handled and tested any tissues from cadavers and animals for the purpose of the paper.

## Results

### Choice of Parameters

To select the suitable parameters for our damage model we use the uniaxial data for the human skin harvested from various locations on the back of a cadaver, presented by Fig. 10 in Ref. 33. Following Ref. 33 we note that *κ* = 0.1404 and *β* = 41° were measured in this particular test. To check if we could inversely identify these parameters from the uniaxial tests only, we run five different cases: in case A, we consider the original GOH model without damage and fix *κ* = 0.1404 and *β* = 41° as the measured values. In cases B–E, we use the damage model Eq. (2), but in case B we keep *κ* = 0.1404 fixed, in case C, we keep *β* = 41° fixed, in case D we keep both *κ* = 0.1404 and *β* = 41° fixed, and in case E, we don’t fix any parameters.

**Figure 5:**
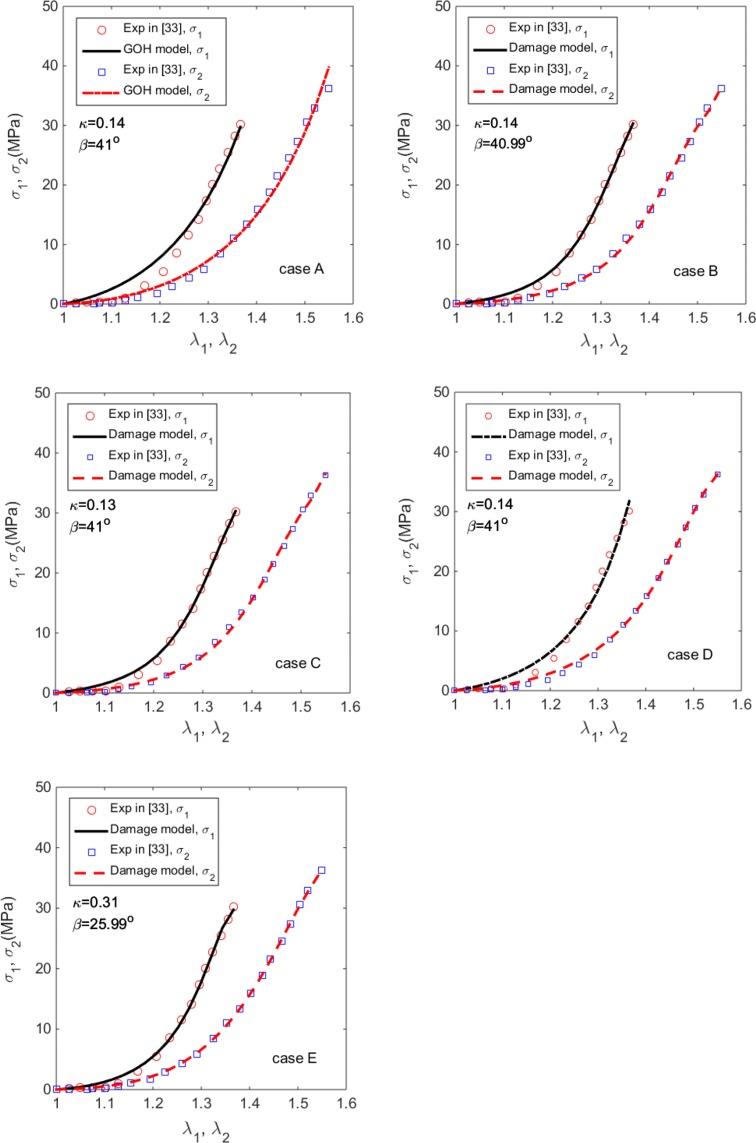
The Cauchy stress-stretch curves from the GOH and the damage models of the human skin samples, compared to the experimental data from, Ref. 33 for case A (the GOH model with *κ* and *β* fixed), case B (damage model with, *κ* fixed), case C (damage model with *β* fixed), case D (damage model with both *β* and *κ* fixed), and case E (damage model with both *β* and *κ* free).

The parameters estimated for these 5 cases are summarized in Table 1. The corresponding stress-stretch curves are shown in Fig. 5. The results show that for case A, although both *κ* and *β* are fixed, the stress-stretch curve computed from the original GOH model is not well matched to the experimental curve, particularly for stretches smaller than 1.3, with the overall error of *ɛ* ≈ 12%. In case D, the extracted seven parameters are nearly the same as those in case B and C. Cases B, C and D show a much better agreement and the estimation of the remaining parameter (*β* or *κ*) also agree with the measured value as shown in Table 1.

**Table 1:** Estimated material parameters for Cases A–E.

Parameters	Results in Ref. 33	A	B	C	D	E
*μ* (MPa)	2.01 × 10^−1^	1.50 × 10^−3^	1.51 × 10^−1^	1.47 × 10^−1^	1.52 × 10^−1^	6.11 × 10^−1^
*k*_1_ (MPa)	24.53	26.38	15.10	14.72	15.25	61.13
*k*_2_	1.33 × 10^−1^	3.58	23.30	22.91	23.23	42.62
*β* (°)	41.00	41.00	40.90	41.00	41.00	25.99
*κ*	1.40 × 10^−1^	1.40 × 10^−1^	1.40 × 10^−1^	1.36 × 10^−3^	1.40 × 10^−1^	3.10 × 10^−1^
*m*	N/A	N/A	3.46	3.30	3.15	4.19
*ζ*	N/A	N/A	3.26	3.27	3.23	3.28
*n*	N/A	N/A	6.30	6.33	6.33	7.12
*ξ*	N/A	N/A	1.10	1.11	1.10	1.06
*ɛ* (%)	N/A	11.73	4.47	4.49	4.50	3.25

The strain rate in these tests was 0.012 s^−1^

In case E, all the material parameters are inversely estimated. This case gives the best curve fitting. However, the fitted parameters are significantly different to the measured values. In other words, it is difficult to estimate both the fibre angle and dispersion accurately using the uniaxial tests alone. Indeed, for most of the cases studied below, information from histology examination is required and a damage model must be used. Since case A shows that even with the measured parameters, using the GOH model without damage does not yield good agreement with the experiment. Even with a damage model, we also need to fix either (*β* or *κ*) as in case B or C, or both as in case D, to obtain the best agreement with the experiment.

### Application to Skins with Damage

We now apply the damage model to a number of other experimental data obtained for animal and human skins. Unfortunately, we do not have direct histology data for these samples, but a range of *κ* = [0.1009, 0.1675] was given for the human skin.^33^ Hence, we have to inversely estimate both *β* and *κ*. First, we consider the swine skin (case F) in Ref. 29. We also consider the uniaxial tests by Ankersen *et al*., for samples harvested from the back (case G), and belly (case H), of an 8-month-old swine.^2^ Finally, we consider a foetal calf back skin test^51^ (case I), samples measured from the human back skin^35^ (case J), and samples measured from rhino back skin dermis^44^ (case K).

In cases F, G, H, I and K, the specimens were harvested from the body, so we choose the spine to be the reference of the mean fibre orientation. However, for case J, the Langer’s line is used as in Ref. 35. We are able to estimate all the parameters for cases H and J from the uniaxial tests for the fibre dispersion parameter *κ* ∈ [0, 1/3]. For cases F, G, I and K, however, a good curve fitting is achieved only when we constrain the value of *κ* in a narrow range [0.1009, 0.1675].^33^ This confirms the importance of the histology input in the model.

**Figure 6:**
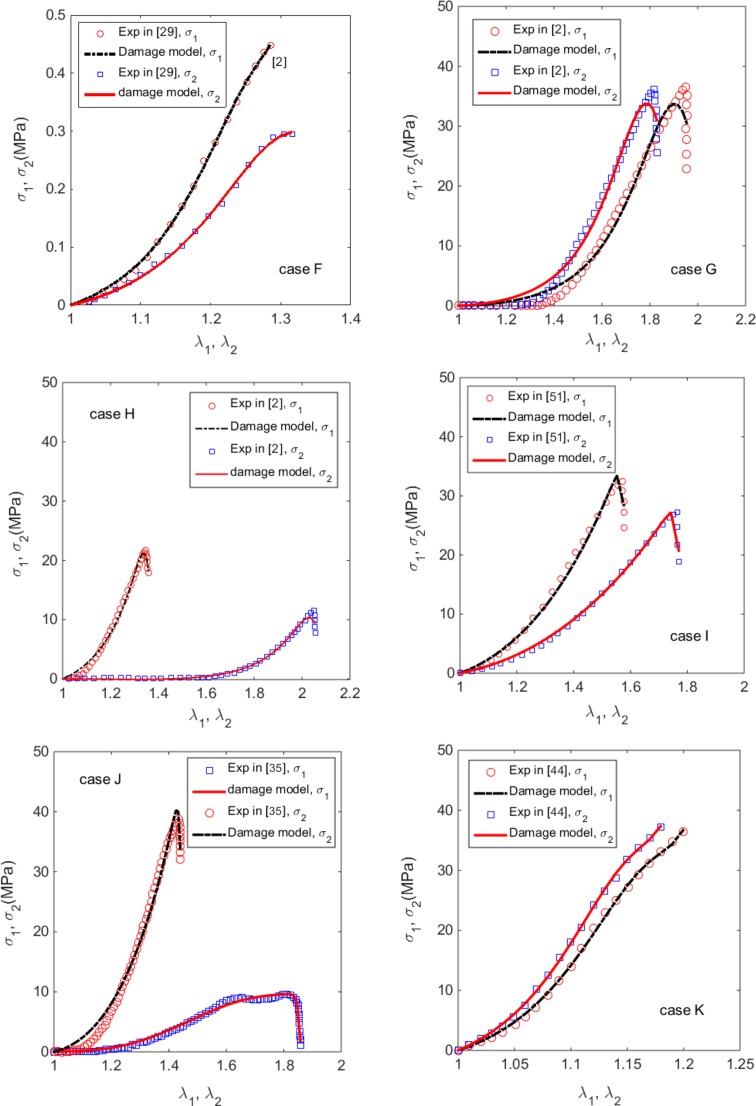
The computed and measured Cauchy stress-stretch curves of animal and human skins, for case F: swine back skin at the strain rate of 2500 s^−1^ in Ref. 29, case G: swine back skin at the strain rate of 0.01 s^−1^ in Ref. 2, case H, swine belly skin,^2^ case I: foetal calf back skin,^51^ case J, human back skin at the strain rate of 0.012 s^−1^,^35^ and case K, rhino back skin.^44^

The estimated parameters for all the cases are listed in Table 2, and the comparisons of the stress-stretch curves are shown in Fig. 6. In all the cases, there are clear damages as exhibited in the experimental data. The stress-stretch curves predicted by the model agree with these experiments well, although we note small discrepancy exists, particularly in case G, where the predicted curves are not as sharp as the experimental data, and the elbow or toe of the predicted stress-stretch relations are less curved compared to the experimental ones.

**Table 2:** Estimated material parameters for cases F–K.

Species	Swine			Bovine	Human	Rhino
Case	F	G	H	I	J	K
Strain rate in tests (s^−1^)	2500	1.00 × 10^−2^	1.00 × 10^−2^	3.00 × 10^−2^	1.2 × 10^−2^	2.20 × 10^−1^
*μ* (MPa)	1.95 × 10^−2^	4.98 × 10^−2^	3.39 × 10^−1^	1.18	5.02 × 10^−1^	3.17 × 10^−1^
*k*_1_ (MPa)	9.57 × 10^−1^	4.97	39.96	12.92	50.22	285.67
*k*_2_	56.33	2.88	7.66 × 10^−1^	1.624 × 10^−1^	1.53	216.27
*β* (°)	42.63	47.98	5.20 × 10^−3^	40.31	6.96 × 10^−2^	46.02
*κ*	1.68 × 10^−1^	1.68 × 10^−1^	2.70 × 10^−1^	1.01 × 10^−1^	2.84 × 10^−1^	1.68 × 10^−1^
*m*	2.36	4.45	1.19	1.39	1.80	2.87
*ζ*	3.11	3.83	3.11	3.27	3.73	3.03
*n*	4.49	6.13	24.30	47.15	56.02	3.71
*ξ*	1.06	1.26	1.12	1.26	1.14	1.03
*ɛ* (%)	2.46	12.18	7.01	6.95	8.02	2.15

### Sensitivity Analysis and Tissue Breaking Criterion

The nine material parameters in the damage model determined from the optimization exhibit different sensitivities to the experimental data. To study this sensitivity, we derive the partial derivatives of strain energy function with respect to the parameters as follows:

6 $$ \begin{aligned} \frac{\partial W}{\partial \mu } = \frac{1}{2}\left[ {\left( {I_{1} - 3} \right) - \frac{{\left( {I_{1} - 3} \right)^{m + 1} }}{{\left( {m + 1} \right)\left( {\zeta - 3} \right)^{m} }}} \right], \, \hfill \\ \frac{\partial W}{{\partial k_{1} }} = \frac{1}{{k_{2} }}\left\{ {\exp (k_{2} A^{2} ) - 1 - \frac{{2k_{2} A^{n + 2} }}{{\left( {n + 2} \right)\left( {\xi^{2} - 1} \right)^{n} }}} \right\}, \hfill \\ \frac{\partial W}{{\partial k_{2} }} = \frac{{k_{1} }}{{k_{2} }}\left\{ {\frac{1}{{k_{2} }}[1 - \exp (k_{2} A^{2} )] - A^{2} \exp (k_{2} A^{2} )} \right\}, \hfill \\ \frac{\partial W}{\partial \xi } = \frac{{4k_{1} nA^{n + 2} }}{n + 2}\frac{\xi }{{\left( {\xi^{2} - 1} \right)^{n} }}, \, \frac{\partial W}{\partial \zeta } = \frac{\mu m}{{2\left( {m + 1} \right)}}\frac{{\left( {I_{1} - 3} \right)^{m + 1} }}{{\left( {\zeta - 3} \right)^{m + 1} }}, \hfill \\ \frac{\partial W}{\partial m} = \left( { - \frac{\mu }{2}} \right)\frac{{\left( {I_{1} - 3} \right)^{m} }}{{(m + 1)\left( {\zeta - 3} \right)^{m} }}\left[ {\ln (I_{1} - 3) - \frac{1}{m + 1} + \ln \left( {\zeta - 3} \right)} \right], \, \hfill \\ \frac{\partial W}{\partial n} = \left( { - 1} \right)\frac{{2k_{1} A^{n + 1} }}{{\left( {n + 2} \right)\left( {\xi^{2} - 1} \right)^{n} }}\left[ {\ln A - \frac{1}{n + 2} + \ln \left( {\xi^{2} - 1} \right)} \right], \hfill \\ \frac{\partial W}{\partial \kappa } = \left( {2k_{1} } \right)\left( {I_{1} - 3I_{4} } \right)A\left\{ {\exp (k_{2} A^{2} ) - \frac{{A^{n} }}{{\left( {\xi^{2} - 1} \right)^{n} }}} \right\}, \, \hfill \\ \frac{\partial W}{\partial \beta } = \left( {2k_{1} } \right)\left( {1 - 3\kappa } \right)A\left( {\frac{{dI_{4} }}{d\beta }} \right)\left\{ {\exp (k_{2} A^{2} ) - \frac{{A^{n} }}{{\left( {\xi^{2} - 1} \right)^{n} }}} \right\}, \hfill \\ \end{aligned} $$

where $$ {{dI_{4} } \mathord{\left/ {\vphantom {{dI_{4} } {d\beta }}} \right. \kern-0pt} {d\beta }} = - \lambda_{i}^{2} \sin 2\beta {\text{ for the specimen }}i, \, i = 1,2. $$

**Figure 7:**
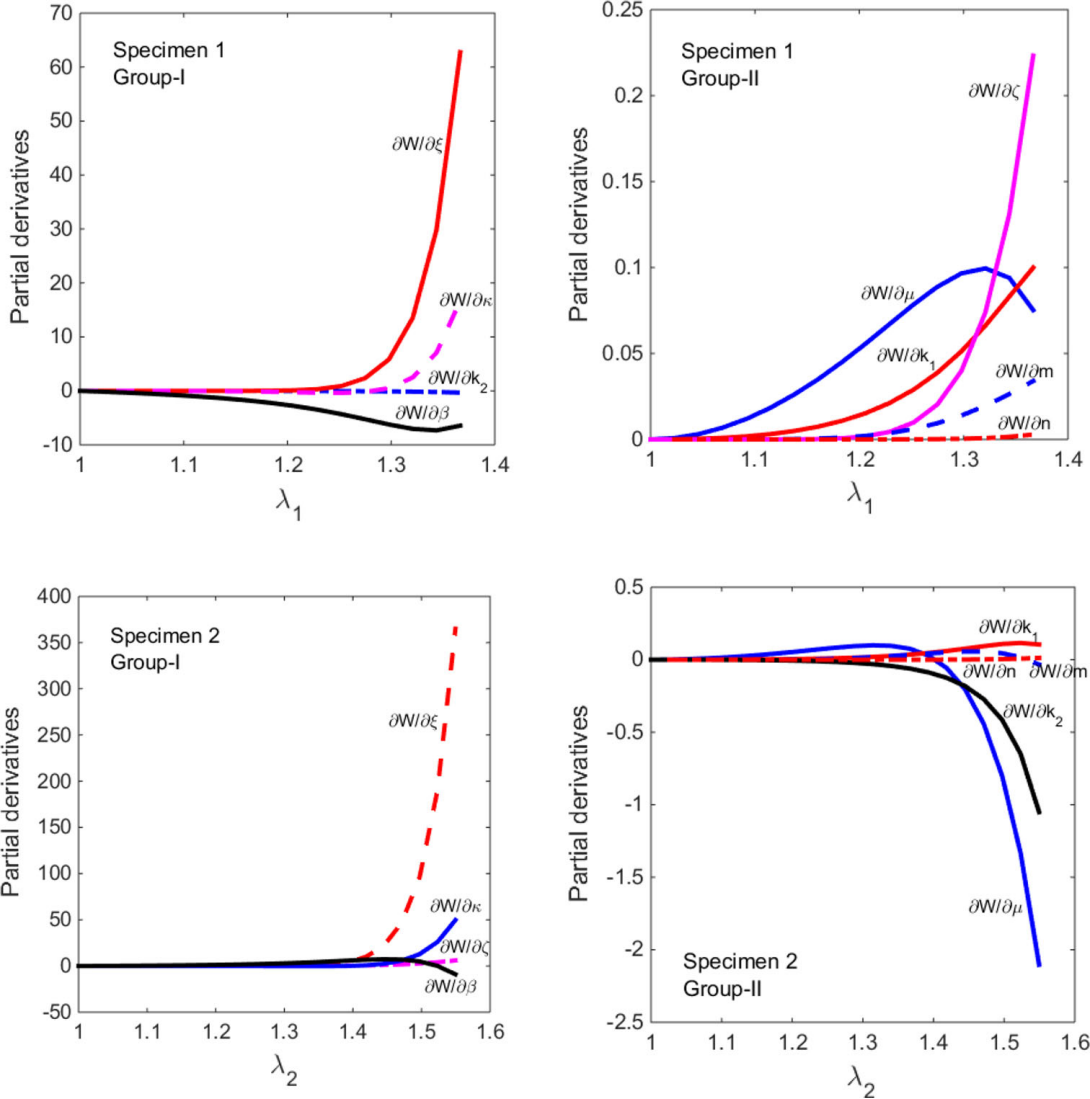
The partial derivatives of the strain energy function plotted against the stretches in Case B for specimen 1 and specimen 2. These are plotted as two groups, with the group-II (∂*W*/∂*μ*, ∂*W*/∂*ζ*, ∂*W*/∂*k*_1_, ∂*W*/∂*k*_2_, ∂*W*/∂*m*) being the orders of magnitude smaller than the group-I (∂*W*/∂*β*, ∂*W*/∂*κ*, ∂*W*/∂*ξ*).

By way of illustration, we plot these nine partial derivatives of the strain energy function in Fig. 7 as functions of stretch *λ*_1_ and *λ*_2_, respectively, based on the parameters of case B (see Table 2). We found that the values of ∂*W*/∂*μ*, ∂*W*/∂*ζ*, ∂*W*/∂*k*_2_, ∂*W*/∂*k*_1_, ∂*W*/∂*m* and ∂*W*/∂*n* (we call these group-II) are orders of magnitude smaller than that of ∂*W*/∂*β*, ∂*W*/∂*κ* and ∂*W*/∂*ξ* (we call these group-I).

Figure 7 also shows that the magnitudes of the partial derivatives increase sharply at the stretches corresponding to the turning points of the stress-stretch curves when damage occurs. From the parameters optimization procedure, we know that the parameters that have larger magnitudes of partial derivative can be determined more accurately than those with smaller values. Hence, the group-I parameters are easier to determine than these in the group-II.

We further rank the significance of the group-I parameters based on the absolute values of their partial derivatives and found that for many cases, e.g. case B, we have

7 $$ \left\{ {\begin{array}{*{20}l} {\xi > \kappa > \beta > k_{2} {\text{ for specimen 1,}}} \hfill \\ {\xi > \kappa > \beta > \zeta {\text{ for specimen }}2.} \hfill \\ \end{array} } \right. $$

We note that the fibre angle is more aligned in the specimen 1 tension direction than that of the specimen 2. Therefore, the fibres are less stretched in the specimen 2. Hence to reach the fibre break limit, a much greater displacement is required for the specimen 2. As a result, there is a possibility that the matrix breaks earlier than the fibres. This may explain the change of rank of the parameter list in (7).

The sensitivity ranking for cases B, F–K is summarized in Table 3. In many cases, we notice that *κ* and *β* consistently occur in the lists of importance. As *ξ* is the parameter associated with the fibre damage, this suggests that the fibre strength plays a crucial role in the mechanical behaviour of skin. Hence, we believe the parameters associated with the partial derivatives in group-I are all fibre-related. These play the dominant roles in the mechanical response of skins compared to these in the group-II.

**Table 3:** Sensitivity ranking of parameters in terms of gradients of strain energy function for Cases B, F–K.

Case	Specimen	Ranking	Break
B	1	*ξ* > *κ* > *β* > *k*_2_	No
	2	*ξ* > *κ* > *β* > *ζ*	
F	1	*ξ* > *κ* > *β* > *ζ*	No
	2	*ξ* > *κ* > *ζ* > *β*	
G	1	*ξ* > *κ* > *k*_2_ > *β*	Yes
	2	*ξ* > *κ* > *k*_2_ > *β*	
H	1	*κ* > *ξ* > *k*_2_ > *ζ*	Yes
	2	*ξ* > *κ* > *k*_1_ > *β*	
I	1	*k*_2_ > *β* > *κ* > *ξ*	Yes
	2	*k*_2_ > *β* > *κ* > *ξ*	
J	1	*κ* > *ξ* > *k*_2_ > *ζ*	Yes
	2	*ξ* > *κ* > *β* > *k*_1_	
K	1	*ξ* > *κ* > *β* > *ζ*	No
	2	*ξ* > *κ* > *β* > *ζ*	

A somewhat different rank lists occur for cases G, H, I and J, in which *k*_2_ also appears. In the extreme case (case I), the rank lists become,

8 $$ \left\{ {\begin{array}{*{20}l} {k_{2} > \beta > \kappa > \xi {\text{ for specimen 1,}}} \hfill \\ {k_{2} > \beta > \kappa > \xi {\text{ for specimen 2}} .} \hfill \\ \end{array} } \right. $$

In cases B, F, H, J and K, *ζ* also appears on the list, presumably because the specimens in these cases were significantly stretched and thus induced the matrix damage also. If the matrix damage is significant in the soft tissue, as shown by cases H and J in Fig. 6, we refer to this as the ductile break. This is opposed to the brittle break where the fibres are damaged first.

**Figure 8:**
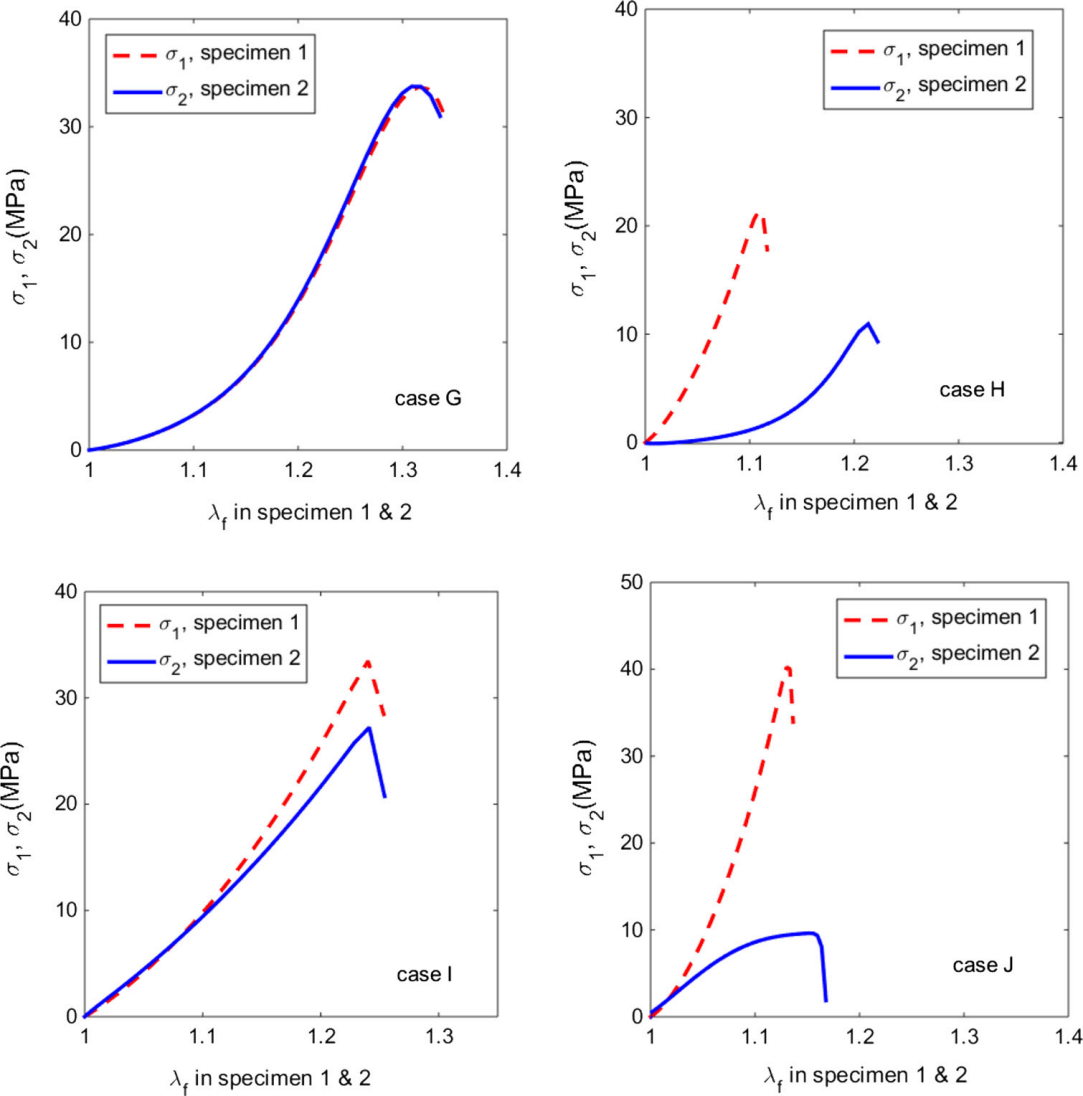
The Cauchy stress components *σ*_1_, *σ*_2_, plotted in terms of fibre stretch *λ*_*f*_. Tissue damage occurs at the stretches when the stresses peak. In cases G, I and J, damage occurs in fibres only since both specimen are damaged at the same fibre stretch. However, in case H, some damage must exist in the matrix as the two samples are damaged at different fibre stretches.

The fibre stretch *λ*_*f*_ at the maximum Cauchy stress is defined as the fibre break limit.^54^ In cases G, H, I and J, there exists a local maximum Cauchy stress in both specimens (Fig. 8), suggesting that the fibres have reached their break limit.

## Discussion

In our damage model for animal and human skins, the material parameters are inversely estimated based on two orthogonal uniaxial tests. We have found that it is difficult to estimate the mean collagen fibre angle and dispersion parameter from the uniaxial data alone.

The sensitivity analysis shows that the mean fibre angle and the dispersion parameter are among the most significant parameters. In general, histological information is required to estimate these two parameters accurately. Indeed, our results show that if one of these two parameters can be measured, or if the range of the dispersion parameter can be provided, then the rest of the parameters can be found so that the model results match the experimental stress-stretch data. Unfortunately, except,^21^,^33^ many experimental studies on skins did not perform histological examinations of collagen orientation and dispersion.

Human and animal skins are viscoelastic since the stress-stretch curves change with the strain rate.^29^^,^ 45 Our model is based on the hyperelastic material assumption. Hence, the estimated fibre parameters agreed with the histological observations only at the lower strain rate. For example, the mean fibre angle and dispersion parameter optimized based on the two uniaxial stress-stretch curves at the strain rate of 0.012 s^−1^ are in agreement with the parameters observed histologically for human skin.^33^ For rat skin, the strain rate threshold that the constitutive response of skin starts to be rate dependent is 0.1–0.3 s^−1^.^45^ Unfortunately, such a threshold of strain rate has not been established for human skin.

The specimens in cases G, I and J were harvested from swine, bovine and human backs, but the specimens in case H was from swine belly. For cases G, I, and J the fibre break limit remains the same in the uniaxial tests of specimen 1 and 2. This implies that the tissue damage is due to the fibre breaks, i.e., these tissues have the brittle break. In case H, however, the fibre break limit occurs at different fibre stretches in the two specimens. This suggests that the damage also occurred in the matrix as otherwise the damage should occur at the same fibre stretch. This type of damage is ductile.

We also plot the Cauchy stresses against a different fibre stretch measure, $\sqrt {I_{4} }$, and the matrix stretch measure, *I*_1_. However, neither $\sqrt {I_{4} }$ or *I*_1_ remains constant in cases G, H, I and J. Therefore, these two invariants are not suitable for use as a breaking criterion.

Although this is the first invariant-based damage model applied to animal and human skins, the limitations of our study are also worth mentioning. Our current model parameters are estimated using the uniaxial test data, since there are very few bi-axial damage tests reported. Nevertheless the model could be better validated with the multi-axial tests in future.

Notably, neglecting viscosity when modelling damage in soft tissues might be a non-admissible over-estimation.^14^ It has been shown that animal skin exhibits plastic deformation and Mullins effect under a cyclic load.^32^ These have not been included in our model. In addition, due to the lack of experimental data, we have also omitted the effect of the residual stress in the model. The bundles of collagen fibres twist and extend to the next deeper observational plane in a helical manner, thus a 3D network is found in rat skins.^39^ The 3D network structure is yet to be considered in the modelling of the skin. Finally, in our work, the skin is modelled as a single layer model. However, the skin has multiple layers.^7^ This should be accounted for in future work.

## Conclusion

We have proposed a new damage model for animal and human skins by modifying the Gasser–Ogden–Holzapfel strain energy function for arterial tissues. This new model describes the softening/damage effects using the Volokh-type power functions and consists of three parameters for the matrix and six parameters for the collagen fibres. The material parameters can be inversely determined based on the uniaxial test data using the optimization method in MATLAB. The model is successfully applied to a variety of skins of swine, human, rabbit and bovine, and results match the experimental stress-stretch curves well. Our sensitivity study confirms that the fibre orientation dispersion parameter, *κ*, the mean fibre angle, *β*, are the most important factors that influence the damage model. In addition, these two parameters can only be reliably determined if some histological information for one for these is provided. We also found that depending on the location of skins; the tissue damage may be brittle (i.e., mostly controlled by the fibre breaking limit), or ductile (due to both the fibre and the matrix damages). Finally, we illustrate that the fibre stretch, which is dependent on the fibre dispersion, is the best parameter for use as the fibre breaking limit.
